# What landscape elements are needed for hospital healing spaces? Evidence from an empirical study of 10 compact hospitals

**DOI:** 10.3389/fpubh.2023.1243582

**Published:** 2023-11-23

**Authors:** Haoxu Guo, Weiqiang Zhou, Wenbo Lai, Lihao Yao

**Affiliations:** ^1^School of Architecture, South China University of Technology, Guangzhou, China; ^2^State Key Laboratory of Subtropical Building Science, South China University of Technology, Guangzhou, China; ^3^College of Coastal Agricultural Sciences, Guangdong Ocean University, Zhanjiang, China

**Keywords:** landscape environment, compact hospital, patient preferences, KANO model, healing property

## Abstract

**Background:**

Modern medical research shows that a rationally planned landscape environment helps patients recover. With the growing number of hospital patients and the tightening of *per capita* medical landscape land, the use of limited landscape resources to serve patients has become challenging.

**Methods:**

This study focused on the landscape environment of 10 hospitals in Guangdong Province, China. Based on the KANO theoretical model, a survey questionnaire was designed and administered to 410 participants. The data were analyzed based on demand attributes, importance, sensitivity, and group differences.

**Results:**

The maintenance requirements were the most important item in the sensitivity ranking. Furthermore, the analysis revealed that the users need a safe, quiet, and private environment, owing to their higher requirements, including visual healing, rehabilitation activities, shading and heat preservation, and medical escort. Moreover, adolescents and older adult patients have common and contradictory environmental needs. For example, the landscape environment should provide both an active space and a quiet rehabilitation environment.

**Conclusion:**

This study evaluates how landscape resources can be better utilized from the perspective of the user and expands the theory of healing landscapes, which has practical implications for hospital renovation and landscape environment strategies.

## 1 Introduction

Many studies have shown that a favorable landscape environment in hospitals contributes to patient recovery (1–3). The landscape environment has become one of the most important evaluation criteria for creating modern hospitals, and its role has changed from an “accessory” to hospital buildings to a “necessity” for system planning (4–6). Currently, the landscape environment of Chinese hospitals is undergoing transformations. First, the *per capita* greenspace rate is decreasing sharply due to the increase in the number of patients. According to statistics, the number of hospital beds in China has increased from 2.4 to 6.7 beds per 1,000 people from 2003 to 2021 and will reach 7.5 beds per 1,000 people in 2030. As of 2022, the average green space rate of the 1,629 grade-A tertiary hospital sites in China is only 22.5%, which is less than the national standard of the “Construction Standards for General Hospitals” in 2021 (the New General Hospitals green space ratio needs to be maintained at more than 35%) (7–9). Second, the expansion of medical facility spaces in hospitals is reducing the land for the landscape environment. The accelerated rate of renewal of the current medical system has led hospitals in high-density cities to sacrifice the landscape environment in exchange for the construction land available for medical facilities. A large amount of landscape environment land has been transformed for medical, ancillary, and transportation functions, which results in shrinking areas of hospital landscape environment, homogeneous space, and a reduction in the number of vegetation and species (10, 11). Third, hospital landscape environment enhancement is caught in the dilemma of unsustainable development. Planners and designers usually adopt a concessionary attitude to confront the current situation of hospital landscape constrictions without a bottom-up renewal design strategy from the perspective of the experience and healing of patients in hospitals (7, 12).

Edward Wilson, in his study Pro-Life, asserts that the human instinct to be close to the natural world, that is, “humans are naturally emotionally attached to natural lifeforms,” is the theoretical origin of the tendency of human beings to have a need for healing (13). Subsequently, researchers found that human preferences for natural colors and biomorphic forms are related to the survival instinct to find food and established a systematic sub-theory for the existence of color and biophilic shape (14). The concept of therapeutic landscapes and gardens has been established in the medical field, giving rise to four major theoretical schools: (1) medical geography, (2) environmental psychology, (3) beneficial environments, and (4) horticultural therapy (5). In addition, the environmental psychology school incorporates two major theories: (1) attention restoration theory (ART), which establishes the four restorative environmental characteristics of distance, range, fascination, and compatibility (5, 15). (2) Aesthetic affect theory (AAT), which draws on the psychoevolutionary theory and establishes relief from physical symptoms, illness, or trauma, as well as relief from coping with stress, as the three characteristics of a healing garden (16). Recently, the wellness theory has been widely recognized in healthcare architecture worldwide and is centered on encouraging the ‘wellness factor’ in hospital design to create a restorative environment for patients (17).

At present, research on the hospital landscape environment focuses on the following three aspects: (1) the influence of the landscape environment on the recovery effect: In the early 19th century, Dr. Nightingale found that increasing the species and area of trees planted in the landscape, improving the ventilation of the environment, and choosing warm and comfortable building materials and colors helped patients’ recovery. With the development and popularization of medical technology, the rate of patients’ recovery and treatment has increased significantly. However, the influence of the landscape environment in medical facilities on long-term inpatients has been neglected, with studies revealing that in hospitals with poor landscape environments, patients are confronted with varying degrees of depression and lower mood during hospitalization and after recovery (6, 18, 19), (2) improvement of the medical model based on the results of psychological studies of the landscape environment: Many scholars, after realizing that the hospital landscape environment affects psychology of patients, conducted empirical studies to gradually confirm the negative impact of the “single biological” medical model in the rehabilitation stage. In the United States, the doctor Engel believes that hospitals and medical programs need to eliminate the negative impact of the medical environment on patients and proposes a new “bio-psycho-social” medical model so that the healing value of the landscape environment is back in public view, and (3) research on the influence of the landscape environment on patient healing (20, 21). Malkin proposed, while designing apartments for the older adult, that a healing environment should have five factors: natural contact, comfortable temperature, positive transfer, social support, free space, and elimination of negative environmental impacts. Empirical research has been conducted based on the five-factor framework and has gradually improved the construction of a multi-dimensional, multi-factor, and multi-level index evaluation system (22–26). Xiaodong Lan conducted virtual reality tests of probiotic indoor environments and found differences in anxiety relief processes and changes in physiological indicators of stress reflection in different age groups (27). Tijsen found that older adult populations face greater challenges in coping with the external environment during the rehabilitation process (28). The above-mentioned studies have demonstrated that extensive landscape plant environments are crucial for patient recovery and that the shaping of healing environments should be incorporated into the health and economic benefits of hospital design. The methodology used in the current study focused on qualitative research, including synthesis reviews, video statistics, participant observation, interviews, scenario mapping exercises, and technical methods using quantitative research (29–32).

Currently, there is a mismatch between the needs of patients and the supply of healing environments in China’s austere hospitals. Such an imbalance between the two is due to the overcrowding of public environments by medical buildings. Studies on healthcare healing environments have mostly used conventional statistical methods, focusing on exploring potential patterns of influence, but have failed to explore the needs that are key for the patients, the priority level of their needs, and specific improvement measures, as well as the relative lack of categorization of the different age groups of the population. Therefore, this study constructs an index model of the demand for hospital landscape from the perspective of users, especially patients, proposing a planning strategy for austere hospitals to make use of the limited landscape environment to better serve users of different ages and groups through qualitative research, which expands the relevant theories and specific technical methods of the healing environment.

## 2 Study design

This study establishes a two-dimensional cognitive model of the satisfaction status of the demand characteristics of the landscape environment and the degree of satisfaction of the user group using KANO theory. This theory was proposed by the Japanese Professor Noriaki Kano in 1984, inspired by Herzberg’s two-factor theory, and was mainly applied to product development and optimization in the early stages of its birth. The KANO two-dimensional model aims to set up positive and negative questions by the same requirement type, that is, the change in respondents’ satisfaction when having and not having, and to determine the belonging requirement type by quantitative calculation to assist management decisions. Current KANO research is focused on three major areas: (1) theoretical research to optimize the quantitative statistical methods for each quality attribute of the KANO model (33); (2) practical areas that are widely used in various processes such as product development, production, sales, and promotion to improve the satisfaction of the user group (34, 35); (3) interdisciplinary studies combining with environmental behavior, psychology, sociology, and other disciplines to perform multiple types of demand analysis, such as Chen, Yao, and Lee applied the KANO model to conduct service demand classification studies on urban riverbanks, suburban mountainous areas, urban parks, pedestrian streets, and other recreation area environments, to develop types of services to enhance tourist satisfaction (36, 37).

Fernando used the KANO model to evaluate the quality of healthcare services in Peruvian public–private partnerships with health institutions and concluded that patients have a need for service interaction experiences (38). Rodrigues de Vasconcelos synthesized a KANO model with a balanced score card to tap into the tendency of patient satisfaction in a Brazilian public hospital, improving patient adherence indicators for treatment (39). Lujie Deng collected data from 300 users for the hospital signage system at Guangzhou Hospital, which ultimately reflects the functional requirements (40) of digital intelligence and regional culture. The above studies show that the KANO model has a wide range of applications in hospital service quality improvement and is also applicable to the study of healing environments. The strengths of the hospital healing environment need to be studied through the KANO model, including: (1) a two-dimensional assessment of the importance of environmental needs, both positive and negative, for all environmental user groups, especially patients, reducing subjective misjudgments by respondents to a one-way question. (2) When hospital landscape environment modification fails to meet the needs of a variety of users, the prioritization of needs can be quickly identified, with the first priority being to meet core needs (41). (3) The KANO model can be combined with research tools, such as AHP, QFD, and FIE, which can continue to expand the depth of research in hospital healing environments (39, 42).

**Figure 1:**
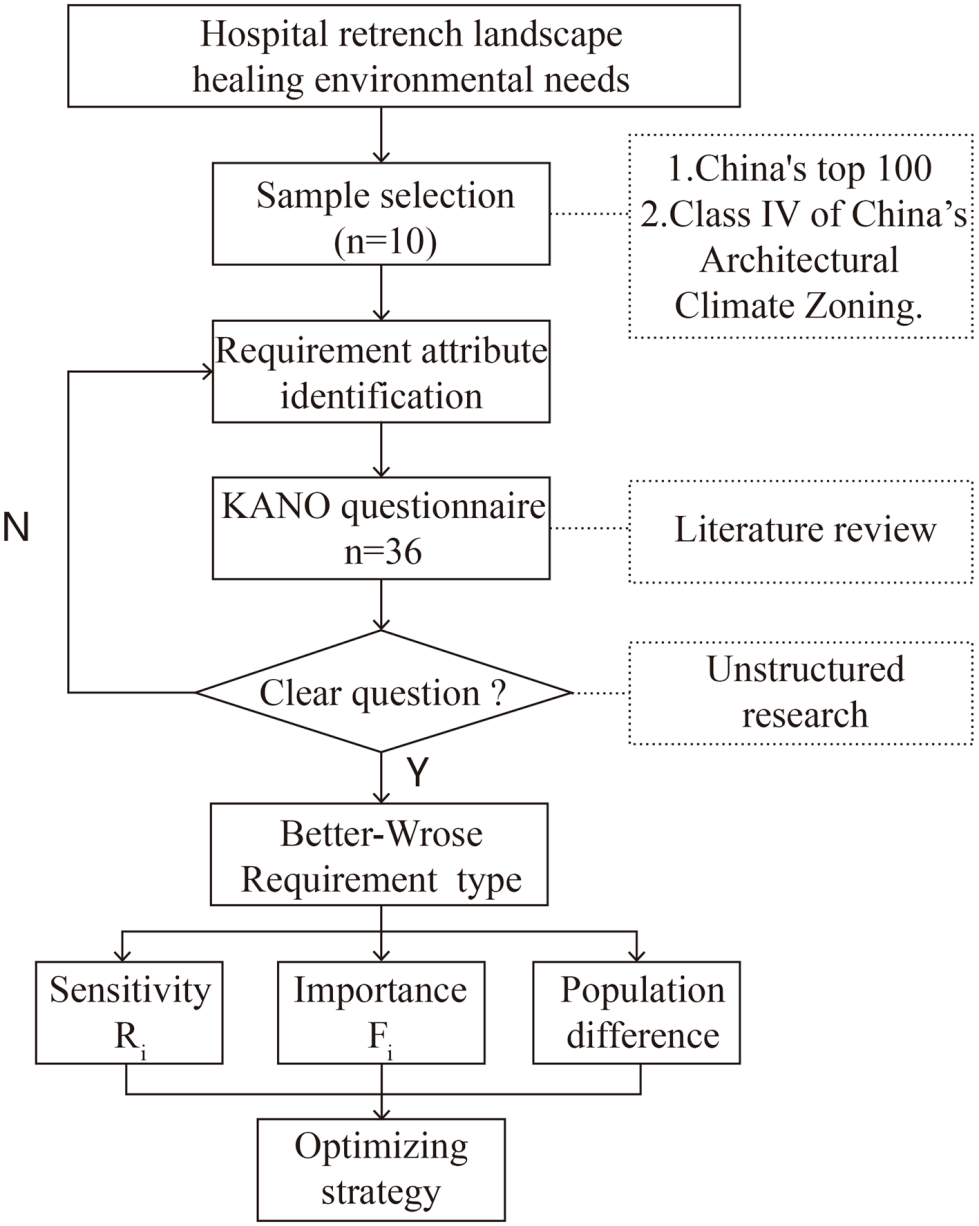
Study design.

The technical route of the research first establishes a composite evaluation index system by identifying demand attributes, sets up a KANO model demand questionnaire corresponding to them individually, and determines whether the content of the questionnaire is comprehensive and clear through preliminary research. Second, the results of the data obtained from this research were classified into demand types for the first time. Finally, secondary classification and evaluation of the need types were conducted by calculating the importance, sensitivity, relative weight, and difference in each index demand (Figure 1).

### 2.1 Study sample

The Fudan edition of the “Top 100 Chinese Hospitals in 2022” is based on the methodology of the American Best Hospitals Ranking Specialist Reputation Assessment, in which the quality of hospitals depends on the authority and dedication of the reviewing experts and is fully recognized by medical units, doctors, and patients. To reduce the differences in the needs of users for healing environments in different geographic and climatic environments, 10 studies selected 10 hospitals as research objects in the Class IV hot summer and cold winter areas of China’s Architectural Climate Zoning. The 10 selected hospitals were all located in an urban center. From the on-site research, it can be seen that seven of the hospitals are still in a state of expansion and reconstruction within the limited land. Their average building density reaches 43% and is higher than the national quartile level of 35%; the average green space rate is only 15%, which is lower than China’s construction standard of 25%; and the average plot ratio of 10 hospitals is 4.07. However, sufficient public space provides conditions for shaping the natural environment, and the greenspace area is one of the most important indicators of the green environment of hospitals. The greenspace ratio is one of the most important indicators of green environments in hospitals. Pakzada, Osmonda, and Corkery’s study of the Green Infrastructure Performance Assessment Framework (GIPAF) model focused on the interactions between human health and urban ecosystems, including the provision of outdoor activities and green space as key indicators (43). Hui Dang and Jing Li evaluated the esthetic value of urban green ecological landscapes by using multi-optical remote sensing to calculate the green space rate index, and the integration of indicators proved that population health and the perception of urban green space landscapes cannot be ignored (44). The 10 samples selected for this study reflect the characteristics of austere hospitals in terms of building density and greenspace ratio, which are representative of the study (Supplementary Table S1).

### 2.2 Setting of the research questionnaire

#### 2.2.1 Factors

Currently, research on healing landscapes involves many disciplines, such as landscape, psychology, sociology, and management, with a comprehensive set of evaluation indicators not yet established. Eliane and Layla used machine learning techniques and regression analysis to determine the relationship between the physical environment of the ward and patient health and found that spatial comfort, safety, autonomy, and self-happiness had a strong influence (45). Karin conducted a healing environment review study in which only 30 out of more than 500 relevant influences were found to have significant effects that could be included in the evaluation criteria, with positive effects in terms of sunlight, air, and location, and differences in the degree of interventions for multiple stimuli such as sound, nature, and television (46). Jody and Woo-Hwa conducted an evidence-based design of healing landscapes, combining 61 healthcare staff empirical studies to uncover landscape preferences such as private spaces, landscaped paths, garden benches, and stream landscapes that are conducive to soothing healthcare work stress (47). Fouad performed a quantitative analysis of the psychological preferences of 148 patients in a post-use evaluation study in a general hospital and revealed that patient satisfaction was positively correlated with space appearance, comfort control, privacy environment, and pleasant healing environment, while age and education were negatively correlated (48). Morales, J. Van, in a study of 798 studies, tapped the positive factors of the physical environment in the rehabilitation process, where security and privacy, clean and comfortable, controlled space, and humanistic care were positive factors for patients and families, whereas environmental comfort, room functionality, and technical support were important factors for staff (49). Patrick, Timothy, and Mukaddes, in their study of therapeutic environments for patients with Alzheimer’s disease, found that rehabilitation exercises, sensory stimulation, mental mapping, shaded and sheltered spaces, as well as security maintenance, had varying degrees of positive effects on patients (50). Stephen’s quantitative systematic rating of healing environments in suburban London found that visual and auditory experiences were the most dominant landscape environment sensory response pathways, whereas smell, taste, and thermal environmental perception did not have a significant effect on healing health enhancement (51). Healing landscape evaluation studies have focused on safety and privacy, landscape configuration, ancillary functions, environmental comfort, rehabilitation facilities, and social support. In this study, based on the basic research above, a pilot study was conducted on 10 research samples to understand the specific needs and preferences of user groups in hospital landscapes and the rationality of indicator construction.

Synthesizing the research results and literature review, this study establishes a multilevel scale for healing environment needs, which contains 5 primary indicators and 18 secondary indicators (Table 1). Landscape demand aims to understand the differences in demand for visual, auditory, olfactory, tactile, and gustatory sensory stimuli among user groups. Functional demand aims to understand the difference in the user group’s demand on landscape function configuration, including private space, security protection, and landscape walkway; experience demand aims to understand the difference in the user group’s demand on social activities and interactive participation, including rehabilitation activities, gardening, and interactive experience; comfort demand aims to understand the difference in the user group’s demand on natural ventilation, lighting, heat insulation, noise reduction, including shading, insulation, air volume regulation, and noise control. Maintenance needs aim to understand the differences in the needs of user groups for daily maintenance of the landscape environment, including resting seats, cleaning and maintenance, air purification, and medical escorts.

**Table 1:** Hospital compact landscape environment healing needs index.

Firstly Requirements	Secondary requirements	Specific instructions	Number
Landscape requirements (A)	Visual perception	Planting of ornamental seasonal plants and composite landscaping	A1
	Auditory perception	Play music that helps to soothe the mood	A2
	Olfactory perception	Planting landscape plants with natural fragrance	A3
	Tactile perception	Installation of landscape vignettes that can be touched and felt	A4
	Taste perception	Provide a safe and hygienic source of drinking water	A5
Functional requirements (B)	Private space	Provide a hidden space that allows communication and relax	B1
	Security	Located in a safe and independent area, provides necessary protection measures	B2
	Landscape walkway	Provide walkable garden paths	B3
Experience requirements (C)	Rehabilitation activities	Provide rehabilitative equipment and devices	C1
	Gardening	Provide simple potted plants that can be watered or sown	C2
	Interactive experience	Provide the possibility of scientific research demonstrations and hand work.	C3
Comfort requirements (D)	Sunshade and heat insulation	Provide louvered roofs or wind and rain corridor	D1
	Air volume regulation	Provide wind compensation in the heat and wind shading in high winds	D2
	Noise control	Install natural barriers to reduce noise	D3
Maintenance requirements (E)	Resting seats	Add seats for rest and conversation	E1
	Cleaning and maintenance	Improve the maintenance of the hospital landscape. Keep the facilities clean and hygienic.	E2
	Purifying the air	Control of external emissions of medical gasses and dirt transport gasses	E3
	Medical escort	Medical accompaniment for patients with limited mobility and the older adult	E4

#### 2.2.2 Questionnaire setting

The questionnaire is divided into two parts: the first part is the basic information of the user group, including gender, age, reason for visiting the hospital, frequency of visiting the hospital, visiting department, and length of stay; a total of six items; and the second part is the KANO questionnaire, corresponding to the secondary indicators set at a total of 36 positive and negative questions. Each question has a 5-level scale, and the options on the questionnaire scale correspond to the secondary needs individually (Supplementary Table S2).

The study samples were all located in the type IV seasonal zone, a region with long summers and no winters, high temperatures, and high humidity throughout the year, with mean temperatures above 10°C in January and approximately 25°C in July and a daily difference in mean annual temperatures of 5–12°C. Therefore, the daily and annual temperature differences in the area were small, the results of the study were less affected by the season, and the distribution of the study questionnaire was scheduled for spring. Previous studies have shown that temperature, noise, wind speed, and solar radiation variations have a direct effect on group perception and comfort evaluation, and special attention should be paid to the effects of extreme environmental factors (52–54). To reduce the physical impact of microclimate differences between different samples on the perception of the user group, we conducted a controlled variable study on the perceived microclimate related to comfort and measured the temperature, humidity, light, noise, wind speed, and air quality indicators of each research sample before distributing the questionnaires. The fluctuation of each indicator in the study sample was less than 30%, which enhanced the validity of the questionnaire by controlling for the fluctuation range of the physical environment.

The survey was conducted using a combination of electronic and paper questionnaires and was distributed from February to March 2023, with survey dates selected from Monday to Friday, from 9:00 to 12:00 and 14:00 to 18:00. The survey was conducted through random sampling in the field, and the target population was a user group located in the public landscape of a hospital. The sample size was 10 times the number of items on the KANO scale, and the total number of samples was at least 150. The number of questionnaires distributed to each hospital was 40–50. Therefore, the total number of questionnaires distributed in this study was 410, excluding some missing questionnaires and 22 invalid questionnaires. A total of 388 valid questionnaires were collected, and the statistical values are shown in Table 2.

**Table 2:** Basic information statistics of the research sample.

Respondent information statistics				Site microclimate statistics				
Projects	Category	Quantity	Proportion	Microclimate measurements	Average value	MIN	MAX	SD
Gender	Male	184	47.42%	Noise (dB)	56.2	48.2	66.2	4.61
	Female	204	52.57%	Lightness (LUX)	3,056	2,532	4,217	720
Age	≤20 years old	12	3.09%	Wind speed (M/s)	1.23	0.13	2.54	0.64
	21–40 years old	170	43.81%	Humidity (%)	49.0	31.9	64.3	14.5
	41–60 years old	162	41.75%	Body temperature (°C)	21.3	18.1	25.4	2.8
	≥60 years old	44	11.34%	Tiling temperature (°C)	19.7	11.4	29.7	7.6
Group type	Outpatient	94	24.23%	Vegetation temperature (°C)	18.4	10.2	27.3	5.3
	Inpatient treatment	92	23.71%	TVOC (mg/m^3^)	0.16	0.006	0.19	0.021
	Visiting companion	122	31.44%	HCHO (mg/m^3^)	0.007	0.002	0.012	0.004
	Medical worker	80	20.62%	PM2.5 (ug/m^3^)	27	20	32	4
Length of stay	≤1 h	76	19.59%	CO (ppm)	1	0	3	1
	1–3 h	244	62.89%	CO2 (ppm)	508	405	570	81
	3–6 h	32	8.25%	Sky view factor (%)	55.1	37.6	73.2	14.2
	≥6:0 h	36	9.28%	Overall quality evaluation	Excellent	Good	Excellent	-

Statistical analysis of the groups using the hospital pocket landscape indicated that 44.85% were male users and 55.15% were female users, with equal proportions and no obvious gender tendencies. The age range of the respondents showed a positive distribution, mainly concentrated in the 21–40 and 41–60 age groups, accounting for a total of 85.56%, reflecting that the frequency of use of the young and middle-aged groups was much higher than that of teenagers and older adult groups, and the demands of the latter two groups were easily ignored in the setting of the medical environment. In the group classification, outpatient and inpatient groups account for 47.94% of the total, indicating the extensive demand of patient personnel for hospital landscape environment settings, and the highest percentage of visiting escorts alone, reflecting the high dependence of this group on public environment places in the process of accompanying medical treatment, and the percentage of medical and nursing workers was only 20.62%. The proportion of those who stayed for less than 1 and 1–3 h was 19.59 and 62.89%, respectively, indicating that the medical environment mainly serves the short-stay group and is less likely to meet the needs of the group staying for a long time.

### 2.3 Statistical methodology

According to Noriaki Kano’s theory and methodological tools, the KANO model categorical evaluation scale consists of forward and reverse questions with secondary indicators and generates a five-level quality categorical assessment matrix table consisting of satisfactory, deserved, neutral, tolerable, and unsatisfactory (34, 39–42). The KANO model divides the demand quality attributes into five categories, including attractive quality (A), expected quality (O), must-be quality (M), indifferent quality (I), reversal quality (R), and questionable quality (Q), a total of six items (Supplementary Table S3). When the hospital landscape provides sufficient attractive quality, the satisfaction of the user group increases, and when it is lacking, there is no effect, which is an unexpected surprise: when the expected quality is sufficient, the satisfaction of the user group increases, and vice versa; no increase in satisfaction of the user group when the expected quality is sufficient, and vice versa, a decrease, being an unexpected need; indifferent quality has no significant effect on the satisfaction of the user group regardless of whether they are sufficient. When the reversal quality is sufficient, it will cause dissatisfaction in the user group; otherwise, it will increase, and it belongs to the content to be controlled. Questionable quality is a problem that confuses respondents, and the proportion of this demand should be controlled.

Quantitatively classify and analyze the needs of user groups. For a certain need (i ), the proportion of the demand for a particular group is $\;A_{i},O_{i},M_{i},I_{i}$ and the percentage of the demand is $\;B_{i}$ and $\;W_{i}$ . The formula used is as follows:


$$B_{i}=\frac{\left(O_{i}{\mathrm{+A}}_{i}\right)}{\left(A_{i}{\mathrm{+O}}_{i}{\mathrm{+M}}_{i}{\mathrm{+I}}_{i}\right)}\;\;\bar{B}=\overset{m}{\underset{\mathrm{i=1}}{\Sigma }}B_{i}$$

$$W_{i}=\frac{\left(O_{i}{\mathrm{+M}}_{i}\right)}{\left(A_{i}{\mathrm{+O}}_{i}{\mathrm{+M}}_{i}{\mathrm{+I}}_{i}\right)}\;\;\bar{W}=\overset{m}{\underset{\mathrm{i=1}}{\Sigma }}W_{i}$$

where $B_{i}$ is the rate of satisfaction improvement for the group of users meeting this demand, $\bar{\;B}$ is the mean value of each satisfaction, $W_{i}$ is the rate of decrease in satisfaction for the group of users who do not satisfy the demand, and $\bar{W}$ is the average value of dissatisfaction. Based on the better–worse coefficient values as the horizontal and vertical axes, the demand types can be classified into four quadrants (Supplementary Figure S1). The first is the quadrant X, where $W_{i}$ are greater than their mean values $\bar{B}$ and $\bar{W}$ , making it the “expectation demand” quadrant, which is the landscape environment that should be continuously maintained to set items. In the second quadrant, $B_{i}$ is less than $\bar{B}$ and $W_{i}$ is greater than $\bar{W}$ , which is a “necessary demand” quadrant, for the landscape environment must exist in the content. In the third quadrant, $B_{i}$ and $W_{i}$ are less than the average value $\bar{B}$ and $\bar{W}$ , making it the “irrelevant demand” quadrant, which belongs to the landscape environment that cannot consider the setting content. In the fourth quadrant, $B_{i}$ is greater than $\bar{B}$ and $W_{i}$ is less than $\bar{W}$ , which is the “charm demand” quadrant, which belongs to the landscape environment in the selective optimization of the configuration of the content. Through the importance of each quadrant demand, it is known that the importance of demand is “necessary demand > expectation demand > charm demand > irrelevant demand.”

## 3 Results

The reliability and validity of the questionnaire were tested using the SPSS software. The magnitudes of several test values for the questionnaire are listed in Table 3. The Cronbach’s test values of the overall questionnaire, positive, and negative questions are between 0.8 and 0.9, and the reliability of the questionnaire is at a good level. The Kaiser–Meyer–Olkin measure values of the overall questionnaire for positive and negative questions are between 0.7 and 0.8, and the validity is at a reasonable level. Bartlett’s sphere test has a significance probability of 0.000, which is less than 0.01, with a reasonable correlation.

**Table 3:** Questionnaire validity and reliability tests.

Content	Number of items	Cronbach’s alpha	KMO Metric	Bartlett’s sphericity test
Positive problem (*n* = 388)	18	0.814	0.753	0.000
Negative problem (*n* = 388)	18	0.849	0.819	0.000
Overall problem (*n* = 388)	36	0.858	0.705	0.000

### 3.1 Demand attribute identification

The B–W attribute classification table was obtained using the KANO model 5 × 5 quality classification evaluation matrix (Table 4), in which the questionable demand (Q) accounted for less than 5% of the total, in line with the attribute classification requirements. Among them, the must-be needs (M) are five items in total, including B1 privacy space, B2, security protection, D1 shelter and heat insulation, D3 noise control, and E1 resting seats, which are the environmental configuration that the hospital pocket landscape must have, otherwise it directly affects the satisfaction of the user group; the expected needs (E) are five items in total, including A1 visual perception, A2 landscape walkway, C1 rehabilitation activities, E2 cleaning and maintenance, E3, purified air, and E4 medical escort, which the user group thinks should be available; attractive needs (A) are five in total, including A3 smell perception, A5 taste perception, B3 landscape walkway, C3 gardening planting, and D2 wind control; indifferent needs (I) are three in total, including A2 auditory perception, A4 tactile perception, and C3 interactive experience, for which the user group has no significant demand for such functions.

**Table 4:** Summary of results for B–W attributes of the KANO model (*N* = 388).

Demand	A (%)	O (%)	M (%)	I (%)	R (%)	Q (%)	$B_{i}$ (%)	$W_{i}$ (%)	B–W
A1	21.65	17.01	14.43	45.36	0.52	1.03	39.27	31.94	E
A2	9.28	4.12	1.03	80.93	3.61	1.03	14.05	5.41	I
A3	22.68	10.31	1.55	53.61	11.34	0.52	37.43	13.45	A
A4	8.25	8.76	5.67	65.46	10.82	1.03	19.30	16.37	I
A5	24.74	9.28	6.70	48.45	10.31	0.52	38.15	17.92	A
B1	13.92	18.04	21.65	41.75	4.12	0.52	33.51	41.62	M
B2	21.13	10.31	27.84	38.66	2.06	0.00	32.11	38.95	M
B3	27.32	10.31	1.80	57.47	1.03	2.06	38.83	12.50	A
C1	15.46	20.62	10.31	52.58	1.03	0.00	36.46	31.25	E
C2	18.04	18.04	5.15	57.73	1.03	0.00	36.46	23.44	A
C3	7.73	5.15	1.03	75.77	9.79	0.52	14.37	6.90	I
D1	10.31	20.10	19.59	47.42	2.06	0.52	31.22	40.74	M
D2	23.20	15.46	5.15	55.15	1.03	0.00	39.06	20.83	A
D3	10.82	12.89	19.07	50.00	6.70	0.52	25.56	34.44	M
E1	10.31	16.49	24.7	44.33	3.09	1.03	27.96	43.01	M
E2	14.95	29.38	10.82	42.78	2.06	0.00	45.26	41.05	E
E3	9.79	35.57	13.92	37.11	2.06	1.55	47.06	51.34	E
E4	11.34	47.94	13.40	26.29	0.52	0.52	59.90	61.98	E

### 3.2 Sensitivity analysis

The contents of Table 4 are divided into four quadrants: the mean value of the better coefficient $\bar{B}$ was 0.34, and the average value of the worse coefficient $\bar{W}$ was 0.30. The primary evaluation of demand satisfaction is achieved through the four-quadrant diagrams, but it is not possible to realize the demand priority in each demand quadrant, combined with Yang’s research on perceived importance, sensitivity ( $R_{i}$ ), and weighting ratio ( $F_{i}$ ). The quantitative index of each demand was calculated using Yang’s perception of importance, and the priority of each demand in the same quadrant was compared horizontally using the score value.


(1) $$R_{i}=\sqrt[{}]{B_{i}{}^{2}{\mathrm{+W}}_{i}{}^{2}}$$

(2) $$F_{i}\mathrm{=max}\left[\frac{B_{i}}{\Sigma_{\mathrm{i=1}}^{m}B_{i}\;}\frac{W_{i}}{\Sigma_{\mathrm{i=1}}^{m}W_{i}\;}\right]\;$$

i is a particular demand and $\;R_{i}$ is the sensitivity of the demand (the larger the value, the clearer the sensitivity perception of the user group). $F_{i}$ is the relative weight ratio of the demand, which describes the relative importance of each demand of the user group.

**Figure 2:**
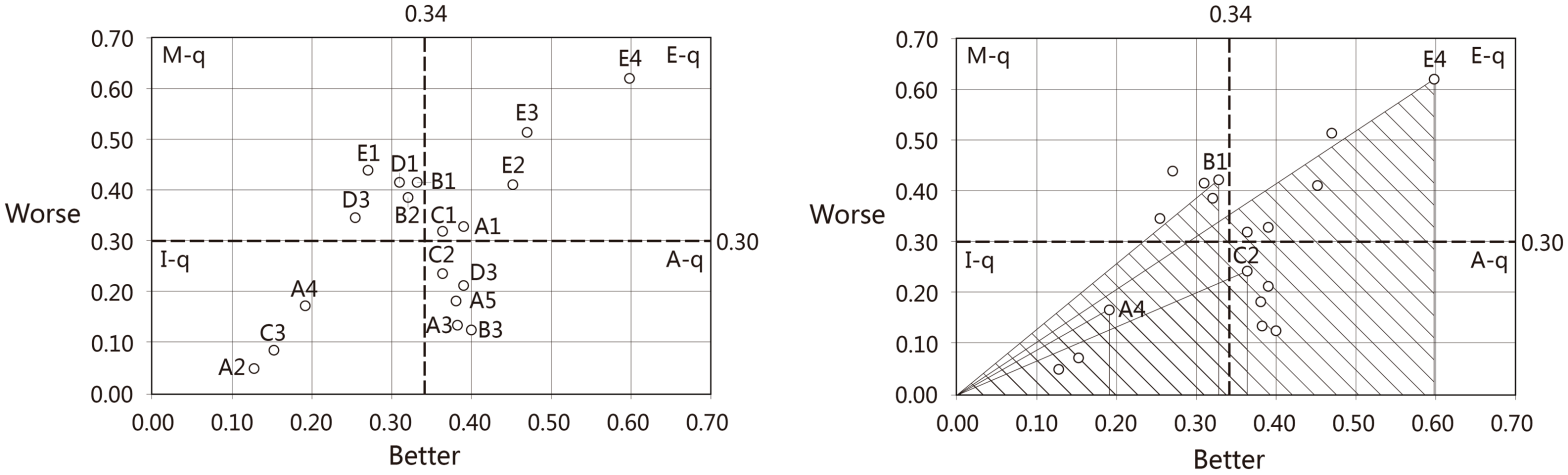
Sensitivity analysis.

According to the quadrant coordinate values (Figure 2), the highest sensitivity in the M-q quadrant is private space (B1), indicating that the lack of relatively independent environmental places in the public landscape to reduce outside interference will significantly reduce the satisfaction of the user group; the highest sensitivity in the E-q quadrant is the medical escort (E4), indicating the importance of having medical personnel present in the public landscape to accompany, which shows a strong correlation with the satisfaction of the user group. The highest sensitivity in the A-q quadrant is gardening (C2), indicating that the provision of certain watering and cultivation experiences has a positive effect on the psychological state of the user group and can be considered for medical landscape systems. The highest sensitivity in quadrants I-q is for auditory perception (A4), indicating that the presence of sound in the landscape environment tends to have a direct effect on user groups, but the demand is not significant.

According to the quadrant classification results (Table 5), we can know that the priority ranking of each attribute requirement is “M-q > E-q > A-q > I-q,” the initial ranking is conducted, and the secondary ranking of each requirement category is achieved through relative weighting. That is, the final result is that the must-be requirements (E1 > B1 > D1 > B2 > D3) are better than the expected requirements (E4 > E3 > E2 > A1 > C1), attractive requirements (D2 > C2 > A5 > B3 > A3), and indifferent requirements (A4 > C3 > A2).

**Table 5:** Demand sensitivity ranking.

B–W	Number	$R_{i}$	$F_{i}$	Order	B–W	Number	$R_{i}$	$F_{i}$	Order
M-q	E1	0.5130	0.1850	1	E-q	C1	0.4802	0.1329	10
	B1	0.5344	0.1732	2	A-q	D2	0.4427	0.1526	11
	D1	0.5133	0.1660	3		C2	0.4334	0.1329	12
	B2	0.5047	0.1517	4		A5	0.4215	0.1455	13
	D3	0.4289	0.1186	5		B3	0.4079	0.1508	14
E-q	E4	0.8619	0.3841	6		A3	0.3977	0.1401	15
	E3	0.6964	0.2635	7	I-q	A4	0.2531	0.0372	16
	E2	0.6111	0.2049	8		C3	0.1594	0.0206	17
	A1	0.5062	0.1542	9		A2	0.1506	0.0198	18

### 3.3 Importance analysis

The data for each demand sub-item under primary demand $R_{i}$ were developed (Table 6). According to the ranking of the mean value of the primary needs, we can see that maintenance management (67.06%) > functional planning (47.51%) > comfort enhancement (46.16%) > experience participation (35.77%) > landscape healing (34.58%), reflecting that the importance of maintenance management is much higher than other primary needs in the hospital healing landscape. Among these, the importance of medical escorts (E4) is the most significant, reflecting that the user group needs a certain amount of escort support when they go out during the rehabilitation process. The importance of functional planning (47.51%) and comfort enhancement (46.16%) are similar, and the standard deviation of the importance of each secondary subparagraph is small and fluctuates little, indicating that the user groups have a higher demand for various functional settings and comfort in the medical landscape. The importance of the former private space (B1) is higher, indicating that the user groups also need relatively independent space in the public landscape environment. The latter has a higher importance for shading and heat insulation (D1), indicating that user groups in the public environment have a significantly higher perception of light and heat than of both ventilation and noise. The mean values of landscape healing (34.58%) and experience participation (35.77%) were close to each other and significantly lower than those of the first three, and the standard deviations of the secondary subscripts are larger and fluctuate greatly, indicating that there are some differences in the specific needs of user groups. The former has a higher importance of visual perception (A1), reflecting that the effect of visual stimulation is better than the rest of sensory perception; the latter has a high importance of rehabilitation activities (C1), indicating that the user groups have a higher perception of the medical healing landscape in the public environment than the other two. The higher importance of the latter rehabilitation activity (C1) indicates that user groups have greater demand for rehabilitation facilities to be configured in the public environment of a medical healing landscape.

**Table 6:** Demand importance ranking.

Firstly requirements	Mean (%)	Max (%)	Min (%)	SD (%)	Secondary subordination
Landscape requirements A	34.58	15.06	50.62	12.72	A1 > A5 > A3 > A4 > A2
Functional requirement B	47.51	40.79	53.16	5.11	B1 > B2 > B3
Experience demand C	35.77	15.94	48.02	14.15	C1 > C2 > C3
Comfort demand D	46.16	42.89	51.33	3.69	D1 > D2 > D3
Maintenance requirements E	67.06	51.30	86.19	12.81	E4 > E3 > E2 > E1
Primary demand mean ranking: E > B > D > C > A					

### 3.4 Group variability analysis

The statistical data were divided into three research groups: age, type, and length of stay, and the sensitivity of each secondary index in the top three groups was ranked to analyze their demand preferences (Table 7). The sensitivity rankings of functional planning, experience participation, and comfort enhancement were the same in each group, with the most significant demands for private space B1, rehabilitation activity C1, and sunshade and heat insulation D1.

**Table 7:** Ranking of differential needs of the group.

Group	Category	Landscape healing	Functional planning	Experience participation	Enhanced comfort	Management maintenance
Age	≤20 years old	A2 > A2 > A4	B3 > B1 > B2	C1 > C2 > C3	D1 > D2 > D3	E4 > E1 > E2
	21–40 years old	A1 > A3 > A3	B1 > B2 > B3	C1 > C2 > C3	D1 > D2 > D3	E4 > E3 > E1
	41–60 years old	A1 > A3 > A4	B1 > B3 > B2	C1 > C2 > C3	D1 > D2 > D3	E4 > E3 > E1
	≥60 years old	A2 > A1 > A4	B1 > B3 > B2	C1 > C2 > C3	D1 > D2 > D3	E4 > E3 > E1
Group Type	Outpatient	A1 > A4 > A5	B1 > B2 > B3	C1 > C2 > C3	D1 > D2 > D3	E4 > E1 > E3
	Hospitalization	A1 > A5 > A3	B1 > B2 > B3	C1 > C2 > C3	D1 > D2 > D3	E4 > E1 > E3
	visitation	A1 > A2 > A5	B1 > B2 > B3	C1 > C2 > C3	D1 > D2 > D3	E4 > E2 > E3
	Medical staff	A1 > A2 > A3	B1 > B2 > B3	C1 > C2 > C3	D1 > D2 > D3	E2 > E3 > E1
Length of stay	≤1 h	A1 > A2 > A3	B1 > B2 > B3	C1 > C2 > C3	D1 > D2 > D3	E1 > E2 > E4
	1–3 h	A1 > A2 > A5	B1 > B2 > B3	C1 > C2 > C3	D2 > D3 > D1	E1 > E2 > E4
	3–6 h	A1 > A3 > A5	B1 > B3 > B2	C1 > C2 > C3	D2 > D3 > D1	E1 > E4 > E3
	≥6 h	A1 > A5 > A2	B1 > B3 > B2	C1 > C2 > C3	D2 > D3 > D1	E4 > E1 > E3

Among the ≤20 years and ≥ 60 years age groups, the sensitivity of auditory perception is high, while the sensitivity of visual perception of 21–40 years old and 41–60 years old groups is better than other sensory perception. In terms of functional planning, the ≤20 years old group values the possibility of touring the garden in healing landscape design, while the rest of the age groups value the creation of private space. Regarding management and maintenance, the 21–40, 41–60, and ≥ 60-year-old groups think that purifying air should be an important demand item after medical care accompaniment. From the age group data and on-site interviews, it can be seen that the sensory organs of the adolescent group are still developing; they are full of curiosity about the outside world, and they are looking forward to exploring fresh and unfamiliar surroundings through their limbs. Young and middle-aged groups rely more on vision to receive external information and form feedback, whereas the older adult group is relatively poor in physical condition, is at a disadvantage in functional and mental recovery, often with stage mental disorders, and shows a higher demand for external healthcare accompaniment.

Among the group types, the difference in needs between the outpatient and inpatient patient groups and the visiting and medical care groups focused on auditory perception, the latter being higher than the former, with the former showing some need for tactile perception, A4, and taste perception. In functional planning, the rankings of experience participation and comfort requirements are the same. In terms of management and maintenance, the outpatient and inpatient groups focused on the adequacy of resting and seating facilities, while the visiting and medical care groups examined whether the degree of tidiness was guaranteed. From the group-type statistics and on-site interviews, it can be seen that the outpatient and inpatient groups often rely on public seating and open space to relieve the fatigue of the recovery process because their bodies are in a state of recovery or discomfort, and their poor mental state also reduces their auditory sensitivity. Visiting and healthcare groups believe that a clean hospital environment is not only conducive to the recovery of the patients but also a hygienic safeguard for the work of the visiting doctors.

In the length of stay group, each group was most sensitive to visual perception, and the main difference was that as the length of stay increased, the sensitivity to the demand for smell and taste perception gradually increased. In terms of management and maintenance indicators, an increase in resting seats and medical escorts was also positively correlated with the length of stay. From the stay time statistics and on-site interviews, it can be seen that the crowd in the hospital public environment with an increase in stay time, anxiety, and boredom will gradually accumulate and need to adjust to bad mood through travel or activities. A long stay increases the knowledge of the crowd about an unfamiliar environment and reduces their precautions regarding the surrounding environment. In addition, there is a need for more resting seats in courtyards to ease the physical exertion of long stays.

## 4 Discussion

The object of this study is austere general hospitals, and no category study has been conducted on special specialist hospitals and hospices; however, the medical landscape needs are discussed in terms of universal design for different groups of people of all ages and are therefore applicable in the design of healing environments. By introducing the KANO theory, this study establishes four quadrant models of needs, and this technical route is efficient, targeted, and easy to implement. The main differences between research on hospital healing environments and traditional multifactor mathematical statistics methods are as follows: 1. Taking the needs of the user group as the fundamental starting point, it ranks demand priorities quickly, whereas traditional research methods such as multiple logistic regressions, principal component analysis, and AHP are used to conduct data correlation analysis and determine its potential mathematical and physical laws from qualitative to quantitative levels. 2. With the ultimate goal of improving the satisfaction of the user group, this method can assist management decision-makers and provide data support to maximize the rational utilization of resources when facing a balance of limited resource allocation. 3. In terms of implementation difficulty, the method has a clear technical process, is friendly to researchers in non-statistical disciplines, and the research content is demand-driven, allowing for iterative research in response to the needs of specific projects.

This study has certain limitations. First, the evaluation index system in this study was based on the statistics of the existing healing landscape research literature, and there is no unified consensus on the evaluation system of the hospital healing landscape in the academic field. Therefore, this study failed to achieve a comprehensive, objective, and systematic index construction process, and further optimization is required. Second, in the research stage, the KANO questionnaire needs to set positive and negative questions; if there are many indicators of landscape healing needs, it will directly lead to multiple questions, and the final completion rate of the questionnaire will affect the validity of the data. The KANO model only provides a type of demand study and lacks judgment on the difficulty of implementing measures to meet demand, which reduces the operability of optimizing the hospital healing landscape environment. Furthermore, due to the limitation of controlling the influencing factors and research efforts, the scope of this study focused on the IV hot summer and cold winter areas in the “China Building Climate Zoning,” which failed to comprehensively consider the healing needs of the patients under the influence of different climates and can be performed as a series of research studies.

This study only investigated the healing needs of the hospital landscape environment. The evaluation index system could be optimized by introducing AHP with a team of experts, user groups, and administrative personnel to assist in the evaluation and improve the research questionnaire. Simultaneously, to improve the feasibility of the study, future research can form a KANO-QFD composite model through the quality function configuration and build a quantitative method to satisfy the difficulty of a certain demand.

## 5 Conclusion

In this study, based on the KANO model, a demand questionnaire for the hospital healing landscape environment was developed to analyze positive and negative demand deviations. Four major quality attributes–must-be quality, expected quality, attractive quality, and indifferent quality–were classified by the better–worse coefficient, and sensitivity and relative weight were introduced to derive the demand ranking of each secondary index. The validity and reliability of each questionnaire were statistically significant. The main findings of this study are as follows:

1. The ranking of the mean values of sensitivity to the needs of the user groups (Table 6) showed maintenance management (67.06%) > functional planning (47.51%) > comfort enhancement (46.16%) > experience participation (35.77%) > landscape healing (34.58%). Given the limited resources of the hospital landscape, it is necessary to strengthen maintenance management efforts, maximize functional planning and comfort facilities, and appropriately reduce the items of sensory healing and experimental participation in healing needs.
2. For settings of hospital landscape environment healing, the healing perception level gives priority to setting visual perception landscape elements, followed by olfactory perception, with no obvious demand for preference for auditory perception and tactile perception landscape elements. The functional planning level needs to focus on the balance between the supply and demand of private and open spaces and provide a relatively independent and safe space for the user group. For the hospital landscape with site conditions, consideration can be given to setting up a landscape tour space. At the level of comfort enhancement, special attention should be paid to the configuration of shading and heat insulation facilities, followed by the control of the acoustic environment, and then wind environment control. At the level of maintenance and management, the medical and nursing staff can greatly enhance the satisfaction of all users, while the environment should be kept as neat and clean as possible to reduce the spread of medical odors.
3. For the study of group variability, it can be seen that the needs of different age groups for the healing landscape have particular preferences; juvenile and older adult groups have some commonalities and contradictions, while the needs of young and middle-aged groups are highly overlapping, and the incremental increase in age shows an increase in dependence on medical and nursing accompaniments. In terms of group type, the outpatient and inpatient groups paid more attention to the visual and tactile environmental needs than the visiting and medical groups. In addition, with the increase in time spent in public environments, there is a significant increase in the need to experience the diversity of the environment, resting space, healthcare accompaniment, and olfactory and gustatory senses, and a decrease in the sensitivity of safety and security.

The results of this study can lay the background for the development of healing landscapes in high-density urban spaces, as well as guide the sustainable development of landscapes in hospital ecosystems at a more refined spatial and temporal scale. Moreover, it provides sustainable strategies for progressively tightening the medical landscape. Furthermore, our approach can be extended to similar social ecosystems in small-scale landscape development planning.

## Data availability statement

The original contributions presented in the study are included in the article/Supplementary material, further inquiries can be directed to the corresponding authors.

## Author contributions

HG: investigation, data collection, formal analysis, visualization, writing–original draft, and project administration. WZ: investigation, data collection, formal analysis, visualization, and writing–original draft. WL: conceptualization, funding acquisition, methodology, supervision, and project administration. LY: supervision, methodology, and writing–review and editing. All authors contributed to the article and approved the submitted version.

## References

[1] Wood VJ Curtis SE Gesler W Spencer IH Close HJ Mason J. Medicine. Creating ‘therapeutic landscapes’ for mental. Health carers in inpatient settings: a dynamic perspective on permeability and inclusivity. Soc Sci Med. (2013) 91:122–9. doi: 10.1016/j.socscimed.2012.09.045 23261254

[2] Marcus CC. Therapeutic landscapes. Environmental psychology and human well-being. Amsterdam, Netherlands: Elsevier, p. 387–413. (2018).

[3] Curtis S Gesler W Fabian K Francis S Priebe S. Therapeutic landscapes in hospital design: a qualitative assessment by staff and service users of the design of a new mental health inpatient unit. Environ Plann C Gov Policy. (2007) 25:591–610. doi: 10.1068/c1312r

[4] Gesler W Bell M Curtis S Hubbard P Francis S. Therapy by design: evaluating the UK hospital building program. Health Place. (2004) 10:117–28. doi: 10.1016/S1353-8292(03)00052-2 15019906

[5] Jiang S. Therapeutic landscapes and healing gardens: a review of Chinese literature in relation to the studies in western countries. Front Arch Res. (2014) 3:141–53. doi: 10.1016/j.foar.2013.12.002

[6] Petros AK Georgi JN. Landscape preference evaluation for hospital environmental design. J Environ Prot. (2011) 2:639–47. doi: 10.4236/jep.2011.25073

[7] Verderber S. Innovations in hospital architecture. Abingdon: Routledge (2010).

[8] Ma X. Issues of public medical insurance reform in China. Public medical insurance reforms in China. Berlin: Springer, pp. 61–84. (2021).

[9] Pan J Shallcross D. Geographic distribution of hospital beds throughout China: a county-level econometric analysis. Int J Equity Health. (2016) 15:1–8. doi: 10.1186/s12939-016-0467-9 27821181 PMC5100192

[10] Li X Zhou L Jia T Peng R Fu X Zou Y. Associating COVID-19 severity with urban factors: a case study of Wuhan. Int J Environ Res Public Health. (2020) 17:6712. doi: 10.3390/ijerph17186712 32942626 PMC7558510

[11] Xiao Y Li Z Webster C. Estimating the mediating effect of privately-supplied green space on the relationship between urban public green space and property value: evidence from Shanghai, China. Land Use Policy. (2016) 54:439–47. doi: 10.1016/j.landusepol.2016.03.001

[12] Weisz U Haas W Pelikan JM Schmied H. Sustainable hospitals: a socio-ecological approach. GAIA. (2011) 20:191–8. doi: 10.14512/gaia.20.3.10

[13] Cuthbert P. Managing service quality in HE: is SERVQUAL the answer? Part 1. Manag Serv Qual. (1996) 6:11–6.

[14] Kellert SR Heerwagen J Mador M. Biophilic design: The theory, science and practice of bringing buildings to life. New York: John Wiley and Sons (2011).

[15] Han K-T Ruan L-W Liao L Health P. Effects of indoor plants on human functions: a systematic review with meta-analyses. Int J Environ Res Public Health. (2022) 19:7454. doi: 10.3390/ijerph19127454 35742700 PMC9224521

[16] Din SK-J Russo A Liversedge JJL. Designing healing environments: a literature review on the benefits of healing gardens for children in healthcare facilities and the urgent need for policy implementation. Land. (2023) 12:971. doi: 10.3390/land12050971

[17] Mittelmark MB Bauer GF Vaandrager L Pelikan JM Sagy S Eriksson M. The handbook of salutogenesis. Berlin: Springer (2022). 36121968

[18] Burpee H. History of healthcare architecture. Washington, DC: Integrated Design Lab Puget Sound, 1–3. (2008).

[19] Collins P. The construction of place as aesthetic-therapeutic In: Williams A, editor. Therapeutic landscapes. Abingdon: Routledge (2017). 349–66.

[20] Parekh AK Kronick R Tavenner M. Optimizing health for persons with multiple chronic conditions. JAMA. (2014) 312:1199–200. doi: 10.1001/jama.2014.10181 25133982

[21] Tavakoli HR. A closer evaluation of current methods in psychiatric assessments: A challenge for the biopsychosocial model. Psychiatry (Edgmont). (2009) 6:25–30. PMID: 19724745 PMC2719450

[22] Malkin J. Medical and dental space planning: A comprehensive guide to design, equipment, and clinical procedures. New York: John Wiley and Sons (2014).

[23] Leibrock CA Harris DD. Design details for health: Making the most of design's healing potential, vol. 9. New York: John Wiley and Sons (2011).

[24] Oh A Jang M YJKIoIDJ L. Healing performance shown in international design guidelines for nursing homes. Korean Inst Interior Des J. (2017) 26:43–52. doi: 10.14774/JKIID.2017.26.1.043

[25] Nolte E Pitchforth E Miani C McHugh S. The changing hospital landscape: an exploration of international experiences. Rand Health Q. (2014) 4:1. PMID: 28560071 10.7249/rhq-04-03-01 PMC5396217

[26] Cho M. Evaluating therapeutic healthcare environmental criteria: architectural designers’ perspectives. Int J Environ Res Public Health. (2023) 20:1540. doi: 10.3390/ijerph20021540 36674294 PMC9865628

[27] Lan X Tan Z Zhou T Huang Z Huang Z Wang C. Use of virtual reality in burn rehabilitation: a systematic review and Meta-analysis. Arch Phys Med Rehabil. (2023) 104:502–13. doi: 10.1016/j.apmr.2022.08.005 36030891

[28] Tijsen LM Derksen EW Achterberg WP Buijck BI. Challenging rehabilitation environment for older patients. Clin Interv Aging. (2019) 14:1451–60. doi: 10.2147/CIA.S207863 31496672 PMC6697645

[29] Zadeh RS Eshelman P Setla J Kennedy L Hon E Basara A. Environmental design for end-of-life care: an integrative review on improving the quality of life and managing symptoms for patients in institutional settings. J Pain Symptom Manag. (2018) 55:1018–34. doi: 10.1016/j.jpainsymman.2017.09.011 PMC5856462 28935129

[30] Hung L Phinney A Chaudhury H Rodney P Tabamo J Bohl D. “Little things matter!” exploring the perspectives of patients with dementia about the hospital environment. Int J Older People Nurs. (2017) 12:e12153. doi: 10.1111/opn.12153 28418180 PMC5574000

[31] Hesselink G Smits M Doedens M Nijenhuis SM Van Bavel D Van Goor H. Environmental needs, barriers, and facilitators for optimal healing in the postoperative process: a qualitative study of patients’ lived experiences and perceptions. HERD. (2020) 13:125–39. doi: 10.1177/1937586719900885 32133876 PMC7364789

[32] Anåker A Von Koch L Heylighen A Elf M. “It’s lonely”: patients’ experiences of the physical environment at a newly built stroke unit. HERD. (2019) 12:141–52. doi: 10.1177/1937586718806696 30336696 PMC6637812

[33] Tontini G. Integrating the Kano model and QFD for designing new products. Total Qual Manage. (2007) 18:599–612. doi: 10.1080/14783360701349351

[34] Xu Q Jiao RJ Yang X Helander M Khalid HM Opperud A. An analytical Kano model for customer need analysis. Des Stud. (2009) 30:87–110. doi: 10.1016/j.destud.2008.07.001

[35] Shi Y Peng Q. Enhanced customer requirement classification for product design using big data and improved Kano model. Adv Eng Inform. (2021) 49:101340. doi: 10.1016/j.aei.2021.101340

[36] Chen M-S Ko Y. Using the Kano model to analyze the formation of regional attractive factors of art street in Taichung, Taiwan. J Asian Arch Build Eng. (2016) 15:271–8. doi: 10.3130/jaabe.15.271

[37] Chen M-S Ko Y-T Lee L. The relation between urban riverbank reconstruction and tourism attractiveness shaping-a case study of love river in Kaohsiung, Taiwan. J Asian Arch Build Eng. (2018) 17:353–60. doi: 10.3130/jaabe.17.353

[38] Barrios-Ipenza F Calvo-Mora A Criado-García F Curioso W. Quality evaluation of health services using the Kano model in two hospitals in Peru. Int J Environ Res Public Health. (2021) 18:6159. doi: 10.3390/ijerph18116159 34200305 PMC8201113

[39] De Vasconcelos CR De Carvalho RSM De Melo FJC De Medeiros D. Improving quality in public Health service: an integrated approach to the Kano model and the balanced scorecard. J Nonprofit Public Sector Market. (2023) 35:215–41. doi: 10.1080/10495142.2022.2066598

[40] Deng L Romainoor NH Zhang BJS. Evaluation of the usage requirements of hospital signage systems based on the Kano model. Sustainability. (2023) 15:4972. doi: 10.3390/su15064972

[41] Materla T Cudney EA Antony J. The application of Kano model in the healthcare industry: a systematic literature review. Total Qual Manage. (2019) 30:660–81. doi: 10.1080/14783363.2017.1328980

[42] Yeh T. Determining medical service improvement priority by integrating the refined Kano model, quality function deployment and fuzzy integrals. Int Schl J. (2010) 4:2534. doi: 10.5897/ajbm.9000204

[43] Pakzad P Osmond P. Developing a sustainability indicator set for measuring green infrastructure performance. Procedia Soc Behav Sci. (2016) 216:68–79. doi: 10.1016/j.sbspro.2015.12.009

[44] Dang H Li J. The integration of urban streetscapes provides the possibility to fully quantify the ecological landscape of urban green spaces: a case study of Xi’an city. Ecol Indic. (2021) 133:108388. doi: 10.1016/j.ecolind.2021.108388

[45] Schreuder E Lebesque L Bottenheft C. Healing environments: what design factors really matter according to patients? An exploratory analysis. HERD. (2016) 10:87–105. doi: 10.1177/1937586716643951 27101834

[46] Dijkstra K Pieterse M Pruyn A. Physical environmental stimuli that turn healthcare facilities into healing environments through psychologically mediated effects: systematic review. J Adv Nurs. (2006) 56:166–81. doi: 10.1111/j.1365-2648.2006.03990.x 17018065

[47] Naderi JR Shin W. Humane design for hospital landscapes: a case study in landscape architecture of a healing garden for nurses. HERD. (2008) 2:82–119. doi: 10.1177/193758670800200112 21161926

[48] Mahmood FJ Tayib A. Healing environment correlated with patients’ psychological comfort: post-occupancy evaluation of general hospitals. Sage J. (2021) 30:180–94. doi: 10.1177/1420326X19888

[49] Huisman ER Morales E Van Hoof J Kort H. Environment. Healing environment: a review of the impact of physical environmental factors on users. Build Environ. (2012) 58:70–80. doi: 10.1016/j.buildenv.2012.06.016

[50] Uwajeh PC Iyendo TO Polay M. Therapeutic gardens as a design approach for optimising the healing environment of patients with Alzheimer's disease and other dementias: a narrative review. EXPLORE. (2019) 15:352–62. doi: 10.1016/j.explore.2019.05.002 31230998

[51] Xue F Lau S. Legacy or lifestyle driver: a London study of healing space in contemporary urban environments (2016) 4:20–42.

[52] Zhang Z Lin B Geng Y Zhou H Wu X Zhang CJE. Buildings. The effect of temperature and group perception feedbacks on thermal comfort. Energ Buildings. (2022) 254:111603. doi: 10.1016/j.enbuild.2021.111603

[53] Ren Z Fu Y Dong Y Zhang P He X. Rapid urbanization and climate change significantly contribute to worsening urban human thermal comfort: a national 183-city, 26-year study in China. Urban Clim. (2022) 43:101154. doi: 10.1016/j.uclim.2022.101154

[54] Ren X Li Q Yuan M Shao SJL Planning U. How visible street greenery moderates traffic noise to improve acoustic comfort in pedestrian environments. Landsc Urban Plan. (2023) 238:104839. doi: 10.1016/j.landurbplan.2023.104839

