# Supplementary material for: What landscape elements are needed for hospital healing spaces? Evidence from an empirical study of 10 compact hospitals
Source: Front Public Health. 2023 Nov 23;11:1243582. doi: 10.3389/fpubh.2023.1243582 (PMC10706125; doi:10.3389/fpubh.2023.1243582)
Supplement: Supplementary file 1 [file Data_Sheet_1.pdf]

Supplementary Material

1 Supplementary Tables

Supplementary Table S1. List of study object.

Study object	Site plan		Scenic environment	
H-1: First Affiliated Hospital of Sun yat-sen university	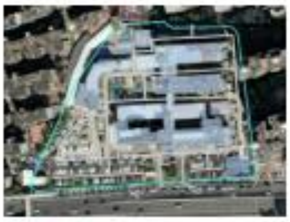		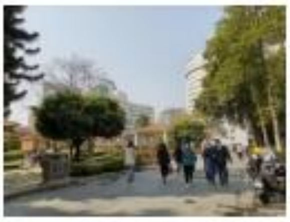	
	Construction time	Density	Greening rate	Far
	1910	52%	7%	4.54
H-2: Nanfang Hospital of Southern Medical University	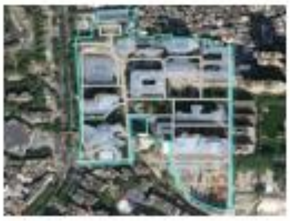		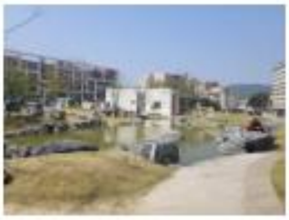	
	Construction time	Density	Greening rate	Far
	1941	35%	16%	2.6
H-3: Sun Yat-sen Memorial Hospital, Sun Yat-sen University	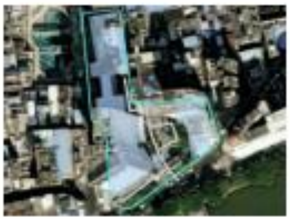		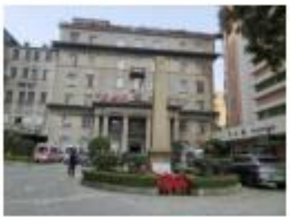	
	Construction time	Density	Greening rate	Far
	1835	51%	7%	6.32

H-4: Guangdong Provincial People's Hospital

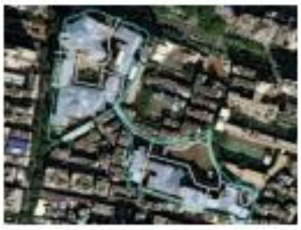

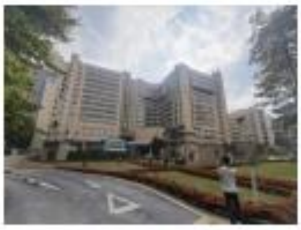

Construction time	Density	Greening rate	Far
1946	45%	12%	4.33

H-5: The First Affiliated Hospital of Guangzhou Medical University

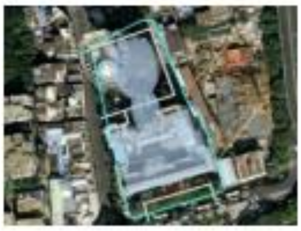

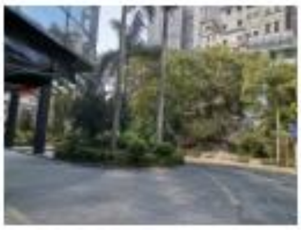

Construction time	Density	Greening rate	Far
1903	54%	14%	6.59

H-6: The Third Affiliated Hospital of Sun Yat-sen University

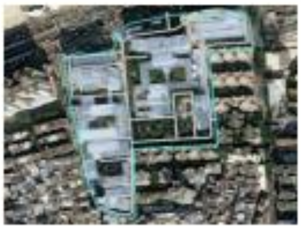

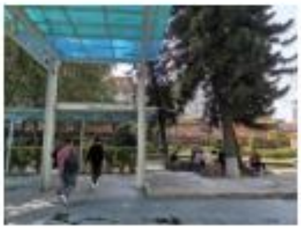

Construction time	Density	Greening rate	Far
1971	42%	19%	3.13

H-7: Zhujiang Hospital of Southern Medical University

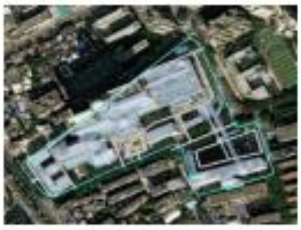

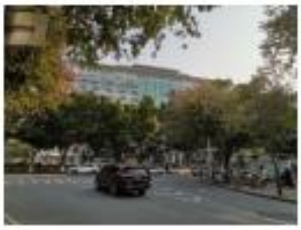

Construction time	Density	Greening rate	Far

	1947	41%	11%	4.23
H-8: Guangzhou First Peoples Hospital	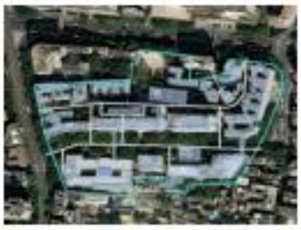		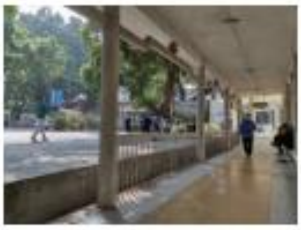	
	Construction time	Density	Greening rate	Far
	1899	39%	24%	3.07
H-9: Shenzhen People's Hospital	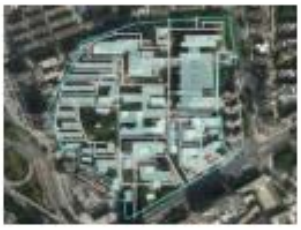		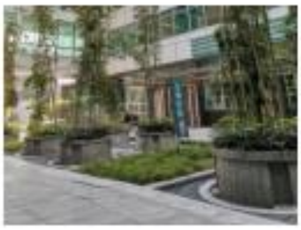	
	Construction time	Density	Greening rate	Far
	1946	36%	19%	2.67
H-10: The Second Affiliated Hospital of Guangzhou Medical University	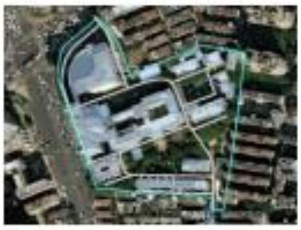		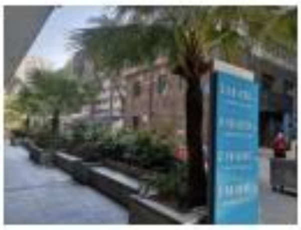	
	Construction time	Density	Greening rate	Far
	1982	35%	21%	3.18

Supplementary Table S2. KANO questionnaire form.

Healing Needs	Visual Perception
Question format	Positive: How do you feel about the increase of ornamental seasonal plants and the diverse landscape combination?<br>Negative: How do you feel about the reduction of ornamental seasonal plants and the diverse landscape combination?
Options	Like it ☐ Expect it ☐ Don't care ☐ Live with ☐ Dislike ☐

Supplementary Table S3. KANO model classification evaluation scale.

Question Type<br>Satisfaction rating		Reverse problem				
		Satisfaction	Deserved	Neutral	Can tolerate	Dissatisfaction
Positive Question	Satisfaction	Q	A	A	A	O
	Deserved	R	I	I	I	M
	Neutral	R	I	I	I	M
	Can tolerate	R	I	I	I	M
	Dissatisfaction	R	R	R	R	O

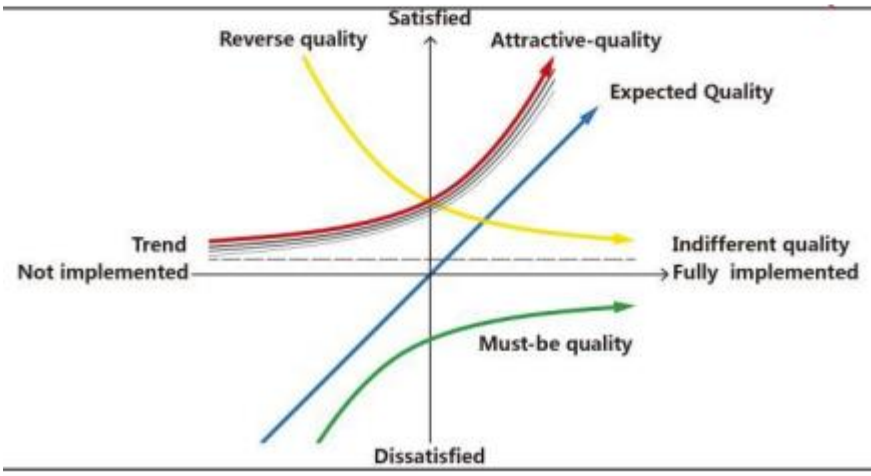

Figure KANO Model

2 Supplementary Figures

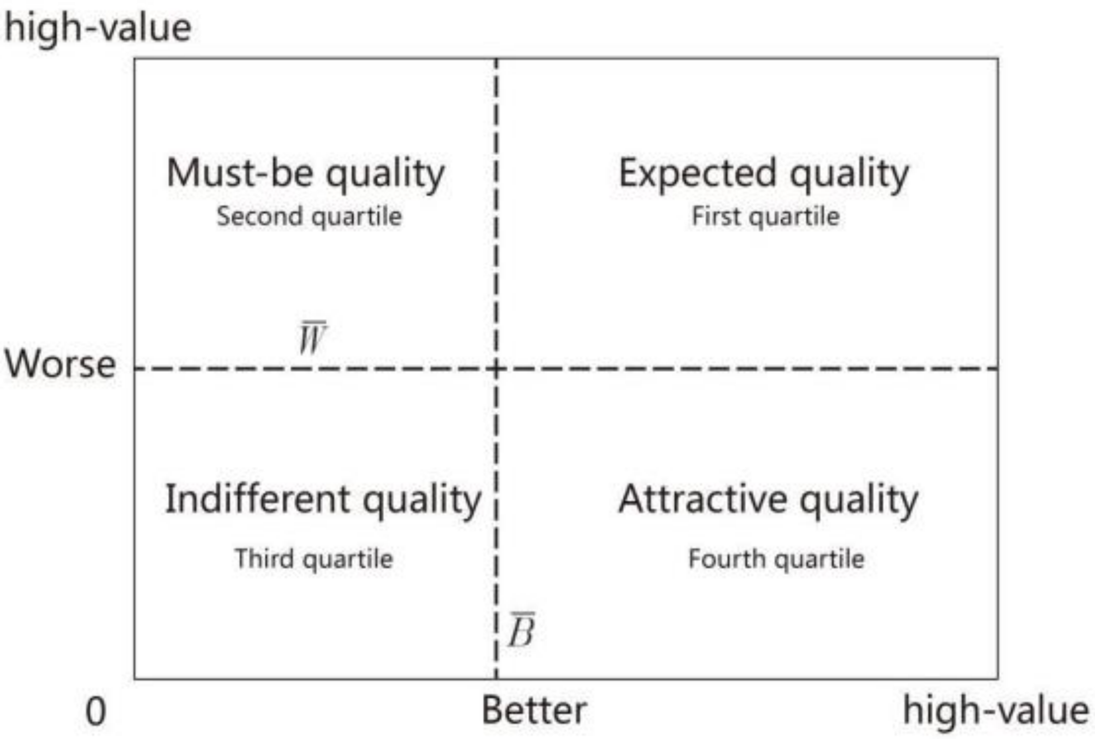

Supplementary Figure S1. Better-Worse Four Quadrant
